# The Impact of Adolescent Alcohol Exposure on Nicotine Behavioral Sensitization in the Adult Male Neonatal Ventral Hippocampal Lesion Rat

**DOI:** 10.3389/fnbeh.2021.760791

**Published:** 2021-11-11

**Authors:** Emily D. K. Sullivan, Liam N. Locke, Diana J. Wallin, Jibran Y. Khokhar, Elise M. Bragg, Angela M. Henricks, Wilder T. Doucette

**Affiliations:** ^1^Department of Psychiatry, Dartmouth-Hitchcock Medical Center, Lebanon, NH, United States; ^2^Geisel School of Medicine, Dartmouth College, Hanover, NH, United States; ^3^Department of Psychological and Brain Sciences, Dartmouth College, Hanover, NH, United States; ^4^Department of Biomedical Sciences, University of Guelph, Guelph, ON, Canada; ^5^Department of Psychology, Washington State University, Pullman, WA, United States

**Keywords:** adolescent alcohol, NVHL, co-occurring disorders, mental illness, smoking, nicotine behavioral sensitization

## Abstract

Nicotine and alcohol use is highly prevalent among patients with serious mental illness, including those with schizophrenia (SCZ), and this co-occurrence can lead to a worsening of medical and psychiatric morbidity. While the mechanistic drivers of co-occurring SCZ, nicotine use and alcohol use are unknown, emerging evidence suggests that the use of drugs during adolescence may increase the probability of developing psychiatric disorders. The current study used the neonatal ventral hippocampal lesion (NVHL) rat model of SCZ, which has previously been shown to have enhanced nicotine behavioral sensitization and, following adolescent alcohol, increased alcohol consumption. Given how commonly alcohol is used by adolescents that develop SCZ, we used the NVHL rat to determine how exposure to adolescent alcohol impacts the development of nicotine behavioral sensitization in adulthood. Male Sprague-Dawley rats underwent the NVHL surgery or a sham (control) surgery and subsequently, half of each group was allowed to drink alcohol during adolescence. Nicotine behavioral sensitization was assessed in adulthood with rats receiving subcutaneous injections of nicotine (0.5 mg/kg) each day for 3 weeks followed by a nicotine challenge session 2 weeks later. We demonstrate that all groups of rats became sensitized to nicotine and there were no NVHL-specific increases in nicotine behavioral sensitization. We also found that NVHL rats appeared to develop sensitization to the nicotine paired context and that adolescent alcohol exposure blocked this context sensitization. The current findings suggest that exposure to alcohol during adolescence can influence behaviors that manifest in the adult NVHL rat (i.e., context sensitization). Interestingly, nicotine behavioral sensitization levels were not altered in the NVHL groups regardless of adolescent alcohol exposure in contrast to prior reports.

## Introduction

Smoking is highly prevalent among patients with serious mental illness and this co-occurrence leads to medical and psychiatric morbidity (Green et al., 1999, 2008; Mallet et al., 2019) as well as an increased mortality risk (Tran et al., 2009; McGinty et al., 2012; Dickerson et al., 2018). Specifically, patients with schizophrenia (SCZ) have higher smoking rates than the general population (de Leon and Diaz, 2005) with lifetime prevalence reported at 60–90% (Volkow, 2009). In one investigation studying patients with SCZ or bipolar disorder, current smokers showed worse cognitive functioning and had poorer functional outcomes than past or never smokers. These effects were observed regardless of diagnosis, however, the patients with SCZ were twice as likely to be smokers compared to those with bipolar disorder (Depp et al., 2015). Moreover, in a recent study, 31% of current smokers were readmitted to a psychiatric hospital within 1 year of discharge compared to 26% of never smokers (Kagabo et al., 2019). Collectively, these studies indicate a correlation between smoking in patients with a serious mental illness and increased psychiatric morbidity and mortality.

The underlying causes of co-occurring mental illness and substance use disorders are largely unknown. However, there is evidence indicating that genetic factors combined with prenatal and/or postnatal developmental insults (including the use of drugs during adolescence; Khokhar et al., 2017), contribute to the development of these disorders. A number of studies suggest cannabis use (Fergusson et al., 2003), and tobacco smoking (Gage and Munafo, 2015; Kendler et al., 2015) may be associated with increased psychotic symptoms. Additionally, for many patients substance use precedes psychosis, with reports finding that substance use rates among patients with first episode psychosis are 30–70% (Abdel-Baki et al., 2017). Thus it is important to study substance use during adolescence and its potential role in contributing to an individual’s risk of developing a psychiatric diagnosis.

One developmental insult used in rats that results in several dysregulated behavioral endophenotypes is the neonatal ventral hippocampal lesion (NVHL). NVHL rats display symptoms resembling those occurring across psychiatric disorders, though they are often used as a model of SCZ (Lipska et al., 1993, 1995; Sams-Dodd et al., 1997; Brady et al., 2010; Gruber et al., 2010; Placek et al., 2013). Moreover NVHL rats self-administer drugs, including nicotine, at a higher rate than normal rats (Chambers and Self, 2002; Berg et al., 2011; Sentir et al., 2020), as well as demonstrate enhanced nicotine behavioral sensitization (Berg and Chambers, 2008). Behavioral sensitization is the progressive increase of drug-induced locomotion with repeated exposure to a drug (Robinson and Berridge, 1993) and is a phenomenon documented in both humans and animals (Kalivas and Stewart, 1991; Robinson and Berridge, 2008). This behavior is indicative of neuroadaptations occurring in motivation related brain regions underlying drug-wanting and craving (Robinson and Berridge, 2008) and can be affected by perturbations occurring in adolescence (McCormick et al., 2004; Mathews et al., 2008; McCormick, 2010; Garcia et al., 2017). Furthermore, cross-sensitization has also been shown, where the repeated exposure of one drug yields sensitization to another drug (Kalivas and Stewart, 1991; Steketee and Kalivas, 2011).

Neonatal ventral hippocampal lesion rats have also been shown to increase alcohol consumption in adulthood after voluntary adolescent alcohol intake (Jeanblanc et al., 2015). Alcohol remains one of the most commonly used drugs by adolescents [Johnston et al., 2020; Substance Abuse and Mental Health Services Administration (SAMHSA), 2020], and as such, combining the NVHL developmental insult with adolescent alcohol exposure can be used to study the complex dynamics between adolescent drug use, SCZ, and increased smoking. In the present study, we used the NVHL rat to determine how exposure to adolescent alcohol affects nicotine behavioral sensitization in adulthood. In humans, alcohol use during adolescence has been linked to increased substance use in adulthood (Ellickson et al., 2003; Grant et al., 2006; Ryan et al., 2019), therefore, we hypothesized NVHL animals with alcohol exposure would demonstrate increased nicotine behavioral sensitization.

## Materials and Methods

### Subjects and Housing

Lactating Sprague-Dawley female rats (*n* = 4) with 10 male pups each were ordered from Charles River (Wilmington, MA, United States) and arrived on the pups’ postnatal day (PD) 2. We specifically chose to use the outbred rat strain Sprague-Dawley in order to maximize the genetic and epigenetic variability, as any behavioral signals would likely be more generalizable to other rats. Additionally, as reports indicate that the prevalence rates of any current tobacco product use is higher in men than women in the general population (Higgins et al., 2015; Cornelius et al., 2020) and in patients with SCZ (Kelly and McCreadie, 1999; Ohi et al., 2018), we used male rats. All rats were housed on a reverse 12-h light cycle with *ad libitum* access to food and water. All experiments were carried out in accordance with the National Institutes of Health Guide for the Care and Use of Laboratory Animals (NIH Publications No. 80–23) and were approved by the Institutional Animal Care and Use Committee of Dartmouth College.

### Neonatal Ventral Hippocampal Lesion Surgery

Neonatal ventral hippocampal lesion or sham (control) surgeries were carried out following previously published guidelines (Chambers and Lipska, 2011). On PD 7 when pups weighed between 15 and 20 *g*, they were anesthetized via hypothermia and then placed on a Styrofoam platform attached to a stereotaxic frame (Kopf Instruments, Tujunga, CA, United States). Half of the pups (NVHL; *n* = 20) were bilaterally injected with 0.3 μl excitotoxic ibotenic acid [10 μg/μl ibotenic acid (Tocris, Minneapolis, MN, United States) in artificial cerebrospinal fluid (aCSF)] into the ventral hippocampi (from bregma: AP −3.0 mm, ML ± 3.5 mm, DV −5.0 mm). The remaining pups (Sham; *n* = 20) were injected with 0.3 μl of aCSF at the same coordinates. After surgery, wounds were closed with surgical glue (VetOne Surgical Adhesive, Boise, ID, United States) and the pups were warmed on a heating pad until their activity level was restored, at which time they were returned to their home cages. In order to control for litter/dam effects, half of each litter underwent the NVHL surgery and the other half was sham-operated. Rats were weaned on PD 21 and housed individually. One sham rat did not recover after surgery.

### Alcohol Drinking in Adolescence

We followed the methods from previously published studies (Jeanblanc et al., 2015; Khokhar and Todd, 2017), but briefly, half of each group [NVHL with alcohol exposure (NVHL AE); sham with alcohol exposure (Sham AE)] was given free access to 10% v/v ethanol (EtOH) in water solution in their home cage for 24 h per day from PD 28–42. Alcohol intake, water intake, and body weights were measured daily and the position of the alcohol and water bottles was alternated each day to prevent development of a side preference. At the end of PD 42, the alcohol bottle was removed, and the rats had access to water only for the duration of the study.

### Nicotine Sensitization

Nicotine behavioral sensitization began on PD 60 and was performed during the active cycle (the time when the animal rooms are dark between 0700 and 1,900 h). Nicotine bitartrate dihydrate (MilliporeSigma, Burlington, MA, United States; 0.5 mg/mL) was dissolved in 0.9% sterile saline, adjusted to 7.4 pH, and administered with a volume of 1 mL/kg bodyweight. Locomotor activity was assessed in four open field arenas (60 cm × 60 cm × 33 cm) located in an animal behavior room, separate from the rats’ housing room. The lights were turned on in the behavior room during nicotine behavioral sensitization (average light intensity was 297.7 lux), and the paradigm was conducted so each round of four animals was comprised of both NVHL and sham rats. The arena used for each individual rat remained consistent throughout the entire experiment and in between each round of four animals, the arenas were thoroughly cleaned.

**FIGURE 1:**
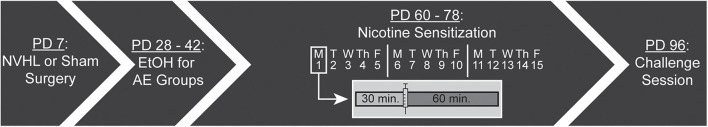
Experimental timeline. NVHL or sham surgery was performed on postnatal day (PD) 7. Half of each group received access to 10% ethanol (EtOH) in their home cage from PD 28–42. Nicotine sensitization began on PD 60 and occurred Monday through Friday for three consecutive weeks (15 sessions). Each session had a 30 min preinjection phase before the rat received a subcutaneous injection of 0.5 mg/kg nicotine followed by a 60 min postinjection phase. After a 2 week washout, all rats had a challenge session on PD 96.

The injection series occurred Monday through Friday for three consecutive weeks (15 sessions). During each session, rats were first placed in the arena for 30 min (i.e., preinjection). After 30 min, each rat was given a subcutaneous (s.c.) injection of nicotine (0.5 mg/kg in 1 mL/kg) and returned to the same arena for 60 min (i.e., postinjection). After the 15th session, rats were given a 2 week washout period where they remained in their home cage. Following the washout period, rats underwent a challenge session where, again, they had a 30 min preinjection period, followed by an injection of nicotine (0.5 mg/kg), and remained in the arena for a 60 min postinjection period (Figure 1). Every pre- and post-injection session was videotaped using a Defeway Security camera system (Shenzhen, China) and analyzed using Noldus EthoVision XT tracking software (Wageningen, Netherlands) for distance traveled (cm), velocity (cm/s), and location within the chamber (i.e., center zone). One NVHL AE rat had to be euthanized after completing the 15 sessions but prior to the challenge session due to seizures. One NVHL no AE rat died before completing the nicotine behavioral sensitization paradigm. Final numbers for the four groups were: NVHL AE = 10; Sham AE = 10; NVHL no AE = 8; and Sham no AE = 9.

### Anxiety-Like Behavior

Anxiety-like behavior was assessed using latency to center, frequency in center, and total duration in center zone for preinjection and postinjection on days 1, 5, 10, 15, and challenge. The center zone (20 cm × 20 cm) was created using EthoVision XT arena settings by dividing the arena floor into nine equal-sized zones. The rat was considered in the center zone if the center tracking point (while using three-point tracking) was within 2 cm of the defined center zone. In sessions where the rat never entered the center zone, the variable latency to center was recorded as the maximum number of seconds for that session (i.e., 1,800 or 3,600 s for preinjection and postinjection, respectively).

### Lesion Verification

**FIGURE 2:**
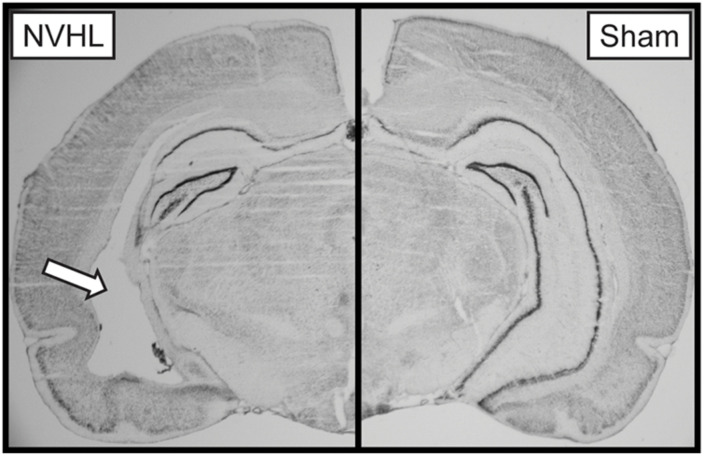
Representative image of a NVHL **(left)** and sham **(right)** brain. The arrow points to the NVHL lesion in the ventral hippocampus.

At the end of the experiment, rats were euthanized by CO_2_ overdose, brains were extracted and flash frozen using 2-methylbutane on dry ice. Tissue was stored at −20°C prior to being sectioned at 40 μm using a Leica Biosystems CM1850 cryostat (Buffalo Grove, IL, United States) and stained with thionin. Lesion size was verified using an AmScope light microscope (Irvine, CA, United States). Lesions include cell loss, cellular disorganization, and ventricle enlargement (Figure 2). NVHL rats with extra-hippocampal damage or unilateral damage were excluded from analysis. One NVHL rat was removed due to an exceedingly large lesion.

### Data Analyses

#### Alcohol Intake

The alcohol intake (g EtOH/kg bodyweight) for each group (NVHL AE or Sham AE) was averaged for each day. A repeated measures analyses of variance (RMANOVA) was used to compare the average alcohol intake between the groups across alcohol exposure time.

#### Nicotine Sensitization

Total distance traveled during preinjection and postinjection was calculated for each day and averaged across groups. To account for individual differences in locomotor activity and to determine the level of nicotine behavioral sensitization, the distance traveled during the first 30 min of postinjection was compared to that days’ preinjection for each rat and expressed as a percentage change. A three-way RMANOVA was run with day (day 1–15) and treatment (preinjection or postinjection) as within-subject factors and group (NVHL AE, Sham AE, NVHL no AE, Sham no AE) as the between group factor. Two-way RMANOVAs were subsequently used to determine group differences in preinjection, postinjection, and percentage change. ANOVAs were used to compare distance traveled between the groups on challenge day. If the assumption of sphericity was violated, the Greenhouse-Geisser correction was used. Any significant effects were further analyzed using Bonferroni *post hoc* tests.

#### Velocity

A three-way RMANOVA was run with day (day 1, 5, 10, 15) and treatment (preinjection or postinjection) as within-subject factors and group as the between group factor. Two-way RMANOVAs were used to compare preinjection and postinjection average velocity between the groups. ANOVAs were used to compare preinjection and postinjection velocity between the groups on challenge day.

#### Anxiety-Like Behavior

Repeated measures analyses of variances were used to compare preinjection and postinjection latency to center, center frequency, and total center duration over days 1, 5, 10, and 15. ANOVAs were used to compare groups during preinjection and postinjection on challenge day. To determine the effect of nicotine on anxiety and to account for an increase in total distance traveled after nicotine, the ratio of center crosses to total distance traveled was calculated for preinjection and postinjection on challenge day. A RMANOVA was used to assess the preinjection and postinjection ratio between the four groups.

## Results

### Adolescent Alcohol Intake

**FIGURE 3:**
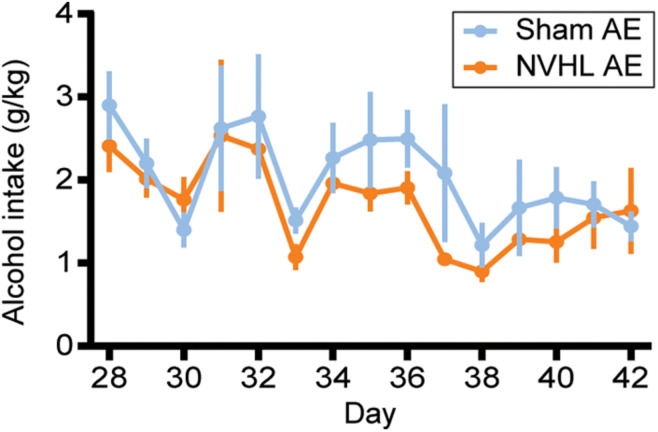
Adolescent alcohol intake. Alcohol Intake (g EtOH/kg bodyweight) from PD 28–42 did not differ between NVHL AE and Sham AE rats. Data is shown as group mean ± SEM.

As shown in Figure 3, RMANOVA revealed that alcohol intake during adolescence did not differ between NVHL AE and Sham AE groups [*F* (1,16) = 0.068, *p* = 0.798].

### Nicotine Sensitization

**FIGURE 4:**
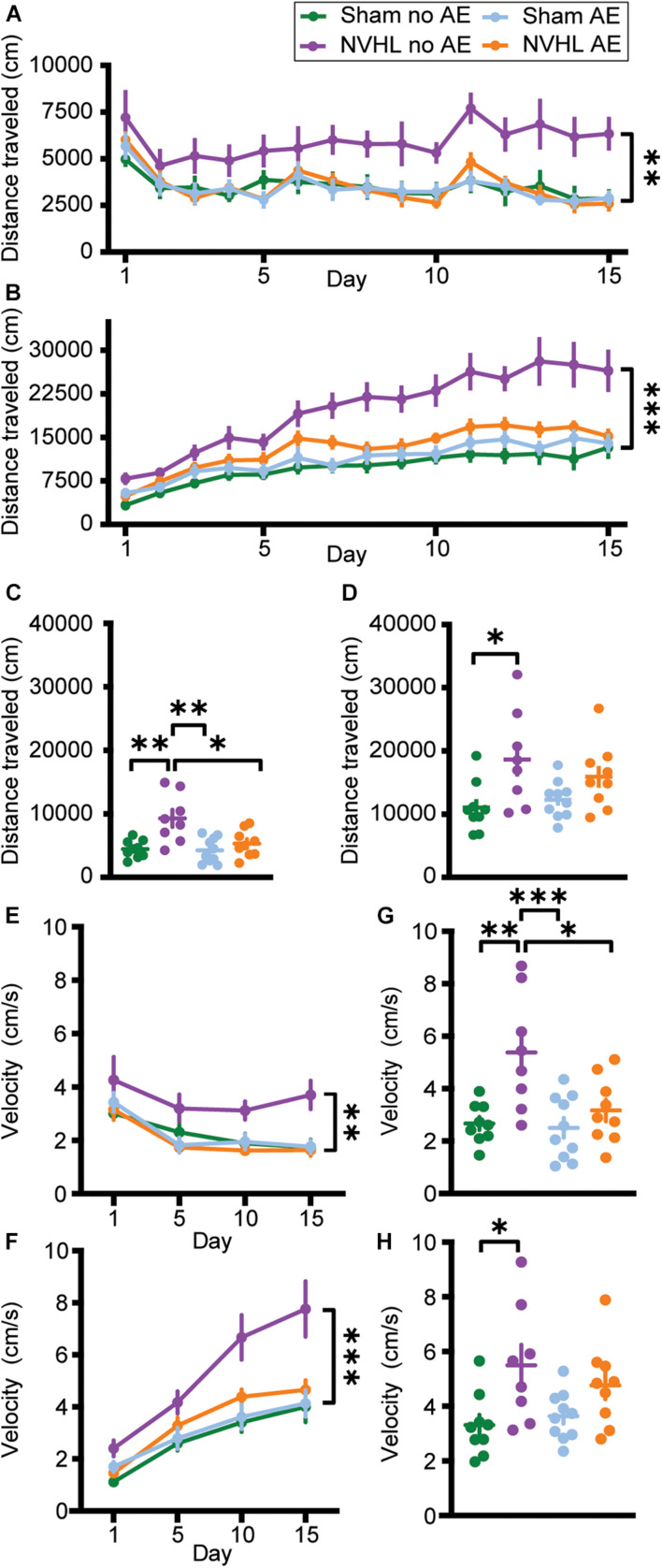
Total distance traveled and average velocity. **(A)** Total distance traveled before an injection of 0.5 mg/kg nicotine across the 15 sensitization sessions. **(B)** Total distance traveled after an injection of 0.5 mg/kg nicotine across the 15 sensitization sessions. The NVHL no AE group showed significantly greater distance traveled compared to the Sham no AE, NVHL AE, and Sham AE groups during both the preinjection and postinjection phases. **(C)** On the preinjection phase of the challenge day, the NVHL no AE group showed significantly more distance traveled than the other three groups. **(D)** During the postinjection phase on the challenge day, the NVHL no AE group only traveled significantly further than the Sham no AE group. **(E)** NVHL no AE rats had significantly elevated average velocity during the preinjection phase on days 1, 5, 10, and 15. **(F)** NVHL no AE rats had significantly elevated average velocity during the postinjection phase on days 1, 5, 10, and 15. **(G)** Average velocity of the NVHL no AE rats remained elevated during the preinjection phase on the challenge day. **(H)** During the postinjection phase, NVHL no AE rats only had significantly increased velocity compared to Sham no AE rats. Data is shown as group mean ± SEM. ^∗^*p* ≤ 0.05; ^∗∗^*p* ≤ 0.01; and ^∗∗∗^*p* ≤ 0.001.

Three-way RMANOVA revealed a significant treatment^∗^day interaction [*F* (3.869,127.671) = 73.656, *p* < 0.001] indicating that distance traveled during postinjection was greater than preinjection, demonstrating nicotine behavioral sensitization. There was also a significant treatment^∗^day^∗^group interaction [*F* (11.606,127.671) = 2.763, *p* = 0.003]. Two-way RMANOVA revealed a significant group effect in distance traveled during the preinjection phase across the 15 nicotine sessions [*F* (3,33) = 4.639, *p* = 0.008; Figure 4A]. Bonferroni *post hoc* analyses showed that the NVHL no AE group traveled significantly further than every other group: Sham no AE (*p* = 0.027), NVHL AE (*p* = 0.021), and Sham AE (*p* = 0.027). A similar pattern emerged when focusing on the postinjection phase. Two-way RMANOVA showed a significant group effect across the 15 nicotine sessions [*F* (3,33) = 10.206, *p* < 0.001; Figure 4B]. Bonferroni *post hoc* analyses showed that the NVHL no AE rats traveled significantly further following nicotine injection than Sham no AE (*p* < 0.001), NVHL AE (*p* = 0.009), and Sham AE (*p* < 0.001) rats.

Looking at the challenge day, an ANOVA indicated a significant difference in distance traveled between groups during the preinjection phase [*F* (3,32) = 7.864, *p* < 0.001; Figure 4C]. Bonferroni *post hoc* showed that the NVHL no AE group traveled significantly further than Sham no AE (*p* = 0.002), NVHL AE (*p* = 0.012), and Sham AE (*p* = 0.001) groups. Additionally, an ANOVA showed a significant difference in distance traveled between groups during the postinjection phase on the challenge day [*F* (3,32) = 4.051, *p* = 0.015; Figure 4D]. Bonferroni *post hoc* analyses indicated that the NVHL no AE group traveled significantly further than the Sham no AE group (*p* = 0.012). However, the NVHL no AE group was no longer significantly different from the NVHL AE or the Sham AE group following the injection of nicotine.

**FIGURE 5:**
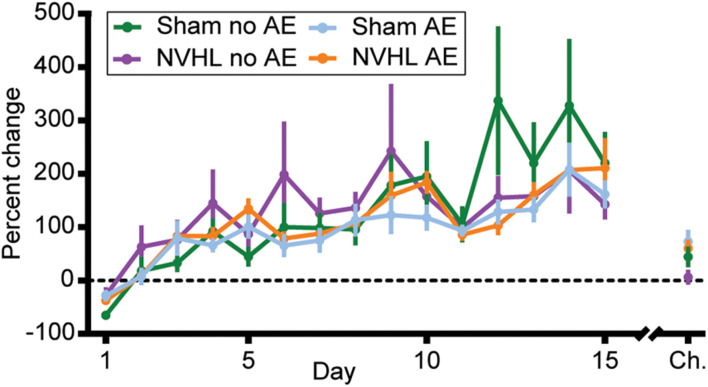
Percentage change in distance traveled. The level of nicotine sensitization was determined by calculating the percentage change in distance traveled from the preinjection phase to the first 30 min of the postinjection phase across each of the initial 15 sessions and on the challenge day for each rat. No group differences were observed but a significant effect of day across the 15 sessions indicates that all groups became sensitized to nicotine. Data is shown as group mean ± SEM.

Since the NVHL no AE group showed significantly greater distance traveled in both the preinjection and postinjection phase, the level of nicotine behavioral sensitization when controlling for any nicotine induced context sensitization was determined by calculating the percentage change in distance traveled from the preinjection phase to the first 30 min of the postinjection phase on each day for each rat. RMANOVA revealed no group differences in the level of nicotine behavioral sensitization across the 15 sessions [*F* (3,33) = 0.380, *p* = 0.768; Figure 5], however, a significant effect of day using the Greenhouse-Geisser correction again indicates that all groups did become sensitized to nicotine [*F* (3.879,128.006) = 16.872, *p* < 0.001]. An ANOVA on percentage change in distance traveled on the challenge day showed no differences between groups [*F* (3,32) = 2.259, *p* = 0.1; Figure 5].

### Velocity

Three-way RMANOVAs revealed significant treatment^∗^day, day^∗^group, and group^∗^treatment interactions. Two-way RMANOVAs showed a group effect in average velocity during the preinjection [*F* (3,33) = 5.165, *p* = 0.005; Figure 4E] and postinjection [*F* (3,33) = 7.949, *p* < 0.001; Figure 4F] phase across days 1, 5, 10, and 15. Bonferroni *post hoc* analyses showed that NVHL no AE rats moved with greater average velocity during both preinjection and postinjection sessions compared to Sham no AE (*p* = 0.027 and *p* = 0.002 for pre- and post-injection, respectively), NVHL AE (*p* = 0.006 and *p* = 0.013), and Sham AE (*p* = 0.023 and *p* = 0.001) groups.

During the challenge day, ANOVA showed a significant difference in average velocity between groups during the preinjection phase [*F* (3,32) = 7.397, *p* = 0.01; Figure 4G] and the postinjection phase [*F* (3,32) = 4.154, *p* = 0.014; Figure 4H]. Bonferroni *post hoc* analyses indicated that during the preinjection phase NVHL no AE rats had greater average velocity than all other groups (Sham no AE [*p* = 0.001], NVHL AE [*p* = 0.018], NVHL no AE [*p* = 0.003]). However, during the postinjection phase, NVHL no AE rats had significantly increased velocity compared to only Sham no AE rats (*p* = 0.025).

### Anxiety-Like Behavior

**FIGURE 6:**
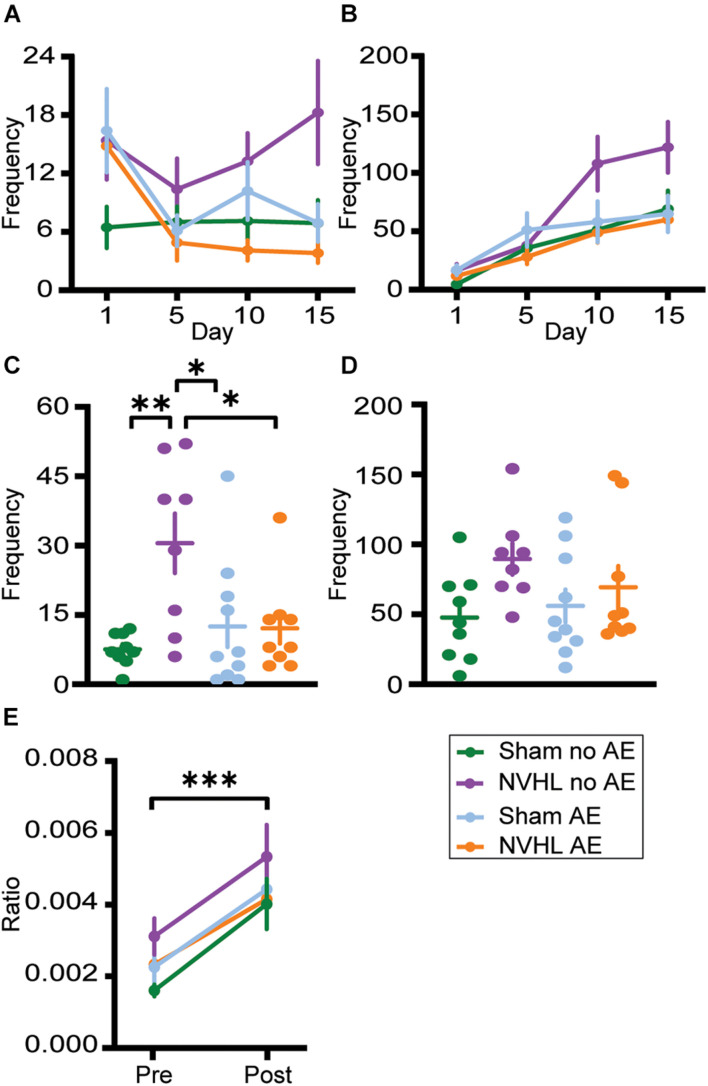
Center zone frequency. **(A)** Frequency of center zone crosses before the injection of nicotine on days 1, 5, 10, and 15. **(B)** Frequency of center zone crosses after the injection of nicotine on days 1, 5, 10, and 15. No significant group differences were observed. **(C)** NVHL no AE rats had significantly increased frequency of center crosses during preinjection on the challenge day. **(D)** Following the nicotine injection on challenge day, no group differences were observed. **(E)** There was a significant increase in the ratio of frequency of center crosses to distance traveled following nicotine on the challenge day with no group differences. Data is shown as group mean ± SEM. ^∗^*p* ≤ 0.05; ^∗∗^*p* ≤ 0.01; and ^∗∗∗^*p* ≤ 0.001.

Repeated measures analyses of variances found no significant differences between groups in latency to center zone (Supplementary Figures 1A,B), frequency in center (Figures 6A,B), and total duration in center (Supplementary Figures 1E,F) in both preinjection and postinjection phases across days 1, 5, 10, and 15.

Similarly, ANOVAs found no significant differences between groups in latency to center zone (Supplementary Figures 1C,D) and total duration in center (Supplementary Figures 1G,H) during preinjection and postinjection on the challenge day. However, there was a significant difference between groups in frequency in center during the preinjection phase on challenge day [*F* (3,32) = 5.518, *p* = 0.004; Figure 6C]. Bonferroni *post hoc* analysis showed that NVHL no AE rats entered the center zone more frequently than Sham no AE (*p* = 0.027), NVHL AE (*p* = 0.028), and Sham AE (*p* = 0.004). Following the nicotine injection on challenge day, the significant differences between groups in center frequency no longer remained (Figure 6D). To assess the effect of nicotine on anxiety-like behaviors and to account for an increase in locomotive behavior after nicotine, the ratio of number of center crosses to total distance traveled was calculated for preinjection and postinjection on the challenge day. RMANOVA revealed a significant increase in the center crosses-to-distance ratio during the postinjection phase [*F* (1,32) = 37.161, *p* < 0.001] with no significant group differences (Figure 6E).

## Discussion

Here, we sought to determine the effects that alcohol exposure during adolescence would have on nicotine behavioral sensitization in the NVHL model of SCZ. The results suggest that adolescent alcohol exposure from PD 28–42 did not alter the amount of nicotine behavioral sensitization. When controlling for baseline differences in distance traveled, there were no differences in the amount of nicotine behavioral sensitization between NVHL and sham rats, regardless of adolescent alcohol exposure. Importantly though, all groups did sensitize to nicotine, as demonstrated by a significant treatment^∗^day interaction in distance traveled and the significant increase in percentage change across the 15 sessions (Figure 5).

The NVHL no AE group showed a significant increase in distance traveled during the preinjection phase of the 15 sessions (Figure 4A), in addition to a significant increase in velocity (Figure 4E). While previous studies have found that postpubertal NVHL rats show spontaneous hyperlocomotion (Lipska et al., 1993; Sams-Dodd et al., 1997), as well as increased locomotor response to a novel environment (Berg and Chambers, 2008), we saw no group differences in distance traveled during the preinjection phase on Day 1. However, we did observe that the NVHL no AE group showed significant increases in distance traveled during the preinjection phase across days once nicotine injections began, suggesting the development of context sensitization, a phenomenon that has previously been reported in the literature in normal rats. Rats treated with nicotine (0.6 mg/kg) for 9 days showed an increase in locomotor activity compared to saline treated animals in the 30 min prior to drug administration (Kosowski and Liljequist, 2005). Similarly, an environment repeatedly paired with nicotine (0.6 mg/kg) acquired the ability to elicit increases in activity even in the absence of nicotine (Walter and Kuschinsky, 1989; Bevins et al., 2001). These data are the first to report that the NVHL rat has enhanced context sensitization, possibly pointing to an increase in the salience of nicotine, and a shift of that salience from nicotine to the context, in this group. Furthermore, it appears that adolescent alcohol exposure impairs the formation of context sensitization, possibly by dampening the salience of nicotine, in the NVHL AE rat. An increase in the salience of nicotine is supported by previous work demonstrating that NVHL rats have increased nicotine seeking behavior during extinction than their sham counterparts (Berg et al., 2013; Rao et al., 2016; Sentir et al., 2020). Thus the current results lend further support to the NVHL rat as a model to better understand SCZ and the increased prevalence of nicotine use.

While additional research is needed to elucidate the exact mechanism, one potential reason for the reduction in context sensitization seen in the NVHL AE group when compared to the NVHL no AE group, may be the impact that alcohol has on developing brain regions. Clinical studies show that alcohol use during adolescence impacts the volume of several brain regions such as the prefrontal cortex (PFC; De Bellis et al., 2005), nucleus accumbens (Thayer et al., 2012), hippocampus (De Bellis et al., 2000; Nagel et al., 2005; Medina et al., 2007), and amygdala (Wilson et al., 2015). Preclinical studies corroborate these findings with alcohol causing numerous anatomical and functional alterations, including decreases in neurogenesis and region-specific brain damage and cell death (Crews et al., 2000; Spear, 2015, 2016). As many of these brain regions play a role in incentive salience, it is possible that disrupted cortical development stemming from alcohol exposure during adolescence dampened the salience of nicotine in the NVHL AE group which prevented the development of context sensitization.

Another neurobiological mechanism that may underlie behavioral changes within the NVHL rat are disruptions in nicotinic acetylcholine receptor (nAChR) function. Extensive literature exists demonstrating that patients with SCZ have disrupted nAChR function and decreased receptor density (Freedman et al., 1995; Breese et al., 2000; Durany et al., 2000; D’Souza and Markou, 2011; Esterlis et al., 2014). These results are supported by a preclinical study showing that NVHL rats have a 12% reduction in nAChR binding in the PFC compared to their sham counterparts (Berg et al., 2015). Furthermore, additional cholinergic alterations exist in this model. *In vivo* acetylcholine release was hyper reactive to both peripheral and local administration of a dopamine (D)_1_ agonist in NVHL rats, and receptor autoradiography showed an increase in muscarinic (M)_1_-like receptor binding sites in the PFC (Laplante et al., 2004a). Another study indicated that tail-pinch stress resulted in a significantly greater increase in PFC acetylcholine release in the NVHL rats, which was subsequently blocked by D_1_ and D_2_ antagonists (Laplante et al., 2004b). Interestingly, nAChRs have been shown to be involved in alcohol-related behaviors where blocking nAChRs partially prevented alcohol-induced locomotor activity (Blomqvist et al., 1992). While some preclinical studies suggest that moderate lengths of alcohol exposure (15–17 days) do not alter nicotinic receptor binding (de Fiebre and Collins, 1993; Ribeiro-Carvalho et al., 2009), chronic alcohol treatment (28 weeks) in rats produced long-lasting reductions in acetylcholine levels, acetylcholinesterase activity, choline uptake, and acetylcholinesterase-positive neurons (Arendt et al., 1988, 1989). Similarly, non-human primates chronically treated with alcohol for 4 weeks had decreased nAChR availability in cortical and thalamic regions (Cosgrove et al., 2010). Though the NVHL AE and Sham AE rats in the current study were only exposed to 14 days of alcohol, there is potential that alcohol exposure during adolescence could alter nAChR function.

Using percentage change as a measure of the amount of nicotine behavioral sensitization, we found that there were no differences between NVHL and sham groups, regardless of whether they received alcohol during adolescence. Our results contrast previously published results showing that NVHL rats (without adolescent alcohol exposure) have enhanced nicotine behavioral sensitization (Berg and Chambers, 2008). A possible explanation for the discrepancies between these studies is the post-weaning housing conditions of the animals. The rats in the current study were singly housed so that alcohol intake during adolescence could be determined for each individual. The rats used in the previously published study (Berg and Chambers, 2008) were pair housed after weaning. Several studies have shown that housing conditions can influence not only the locomotor response to a novel environment, but also the behavioral response to drugs, including nicotine. Rats housed in isolation show increased locomotor response in a novel environment compared to those housed in pairs (Garcia et al., 2017) and those housed in groups (Smith et al., 1997; Cheeta et al., 2001). Furthermore, rats housed in isolation have enhanced sensitization to the locomotor effects of repeated administration of amphetamine (Smith et al., 1997). Additionally, female rats that underwent chronic social stress during adolescence (isolation for 1 h each day and then housed with a new partner) show increased locomotor sensitization in response to amphetamine (Mathews et al., 2008) and nicotine (McCormick et al., 2004). Therefore it is reasonable that isolated housing led to an increase in the nicotine behavioral sensitization of the sham groups, and combined with a potential ceiling effect in the nicotine behavioral sensitization of the NVHL groups, any group differences were masked.

Using measures related to the center zone of the open field arena as a proxy for anxiety-like behaviors (latency to enter the center zone and the duration of time spent in the center zone), we found no differences between NVHL and sham groups, regardless of alcohol exposure (Supplementary Figure 1). The NVHL no AE group did have a significantly increased number of center entrances compared to the other groups, but only during the preinjection phase during the challenge day (Figure 6C). With no other increases in anxiety-like behaviors and the significant increase in both total distance traveled and average velocity, it is likely that the significantly elevated center frequency in the NVHL no AE rats was due to their increased context sensitization. Previous studies assessing anxiety-related behaviors in the NVHL rat have found mixed results based on the method used to measure anxiety. In one study, male NVHL rats demonstrated persistent anxiety as adolescents and adults compared to control rats, spending less time in the central zone of an open field task (Sams-Dodd et al., 1997). However, several other studies found that male and female NVHL rats spend more time in the open arm of an elevated plus maze, suggesting less anxiety (Wood et al., 2003; Beninger et al., 2009). Although the current results found that there were no group differences in anxiety-like behavior using an open field task, future studies assessing anxiety in the NVHL rat should take into account locomotor differences that may confound the results.

In order to assess the effect of nicotine treatment on anxiety-like behaviors and to control for an increase in movement after an injection of nicotine, the ratio of number of center crosses to total distance traveled was calculated for preinjection and postinjection on the challenge day. The significant increase in this ratio during postinjection demonstrates that nicotine had an anxiolytic effect on all groups (Figure 6E). This is consistent with some previous literature showing that 7 days of nicotine treatment (Irvine et al., 2001) or chronic nicotine administered via drinking water (Onaivi et al., 1994) both resulted in anxiolytic effects on elevated plus maze behaviors.

One limitation of the current study was not using both sexes, though there is variability in the literature as to whether a sex difference in nicotine behavioral sensitization exists. Nevertheless, some studies have found that female rats show more locomotor activity in response to nicotine behavioral sensitization (Booze et al., 1999; Harrod et al., 2004), while others find that sex does not have marked influences on this behavior (Kanyt et al., 1999; Ericson et al., 2010). A limited number of studies have used both male and female NVHL rats to assess cognitive abilities (Chambers et al., 1996; Beninger et al., 2009), neurotransmitter release (Beninger et al., 2009), and expression of G-protein coupled receptor kinases (Bychkov et al., 2011). However, to our knowledge, no work has been done exploring sex differences in nicotine behavioral sensitization specifically in the NVHL rat. Another limitation of the current study was the absence of a saline injected group which would serve to control for any handling and injection stress. While this is an important control group, many previous studies have demonstrated that repeated subcutaneous or intraperitoneal injections of saline do not increase locomotor activity (McCormick et al., 2004; Kosowski and Liljequist, 2005; Varvel et al., 2007; Marin et al., 2009; Gomez et al., 2012; Hamilton et al., 2014; Carrara-Nascimento et al., 2020; Trujillo and Heller, 2020). Additional studies corroborate these findings specifically in NVHL rats (Conroy et al., 2007; Berg and Chambers, 2008; Chambers et al., 2013). Therefore, it is highly unlikely that repeated saline injections in our hands would cause behavioral sensitization.

In this study, we found that NVHL rats demonstrated an apparent sensitization to the nicotine paired context, and adolescent alcohol exposure prevented the formation of this context sensitization in the NVHL AE rats. We found that exposure to alcohol during adolescence did not impact the amount of nicotine behavioral sensitization in adulthood. Surprisingly, NVHL rats (regardless of alcohol exposure) did not show increased nicotine behavioral sensitization as had been previously reported, potentially due to post-weaning housing conditions. Nicotine treatment had an anxiolytic effect during the postinjection phase of the challenge day, however, there were no group differences. Future studies could more specifically test the impact of social isolation on nicotine behavioral sensitization and the development of context sensitization in the NVHL rat, as well as expand this work to females.

## Data Availability Statement

The raw data supporting the conclusions of this article will be made available by the authors, without undue reservation.

## Ethics Statement

The animal study was reviewed and approved by Institutional Animal Care and Use Committee of Dartmouth College.

## Author Contributions

ES contributed to planning the experimental design and wrote the manuscript. ES, LL, and EB collected the behavioral data. ES and DW performed the NVHL surgeries and DW contributed to editing the manuscript. AH contributed to data analysis and editing the manuscript. EB contributed to the histological assessment. JK and WD contributed to planning the experimental design and editing the manuscript. All authors read and approved the final manuscript.

## Conflict of Interest

The authors declare that the research was conducted in the absence of any commercial or financial relationships that could be construed as a potential conflict of interest.

## Publisher’s Note

All claims expressed in this article are solely those of the authors and do not necessarily represent those of their affiliated organizations, or those of the publisher, the editors and the reviewers. Any product that may be evaluated in this article, or claim that may be made by its manufacturer, is not guaranteed or endorsed by the publisher.

## Supplementary Material

Supplementary Figure 1 Latency to and duration in center zone. **(A)** There were no significant group differences in latency to center zone during the preinjection phase on days 1, 5, 10, and 15. **(B)** There were no significant group differences in latency to center zone during the postinjection phase on days 1, 5, 10, and 15. **(C)** There were no significant group differences in latency to center zone during preinjection phase on the challenge day. **(D)** There were no significant group differences in latency to center zone during postinjection phase on the challenge day. **(E)** There were no significant group differences in the total duration of time spent in the center zone during the preinjection phase on days 1, 5, 10, and 15. **(F)** There were no significant group differences in the total duration of time spent in the center zone during the postinjection phase on days 1, 5, 10, and 15. **(G)** There were no significant group differences in the total duration of time spent in the center zone during the preinjection phase of the challenge day. **(H)** There were no significant group differences in the total duration of time spent in the center zone during the postinjection phase of the challenge day. Data is shown as group mean ± SEM.
