# Supplementary figures and images for: The Impact of Adolescent Alcohol Exposure on Nicotine Behavioral Sensitization in the Adult Male Neonatal Ventral Hippocampal Lesion Rat
Source: Front Behav Neurosci. 2021 Nov 11;15:760791. doi: 10.3389/fnbeh.2021.760791 (PMC8632551; doi:10.3389/fnbeh.2021.760791)

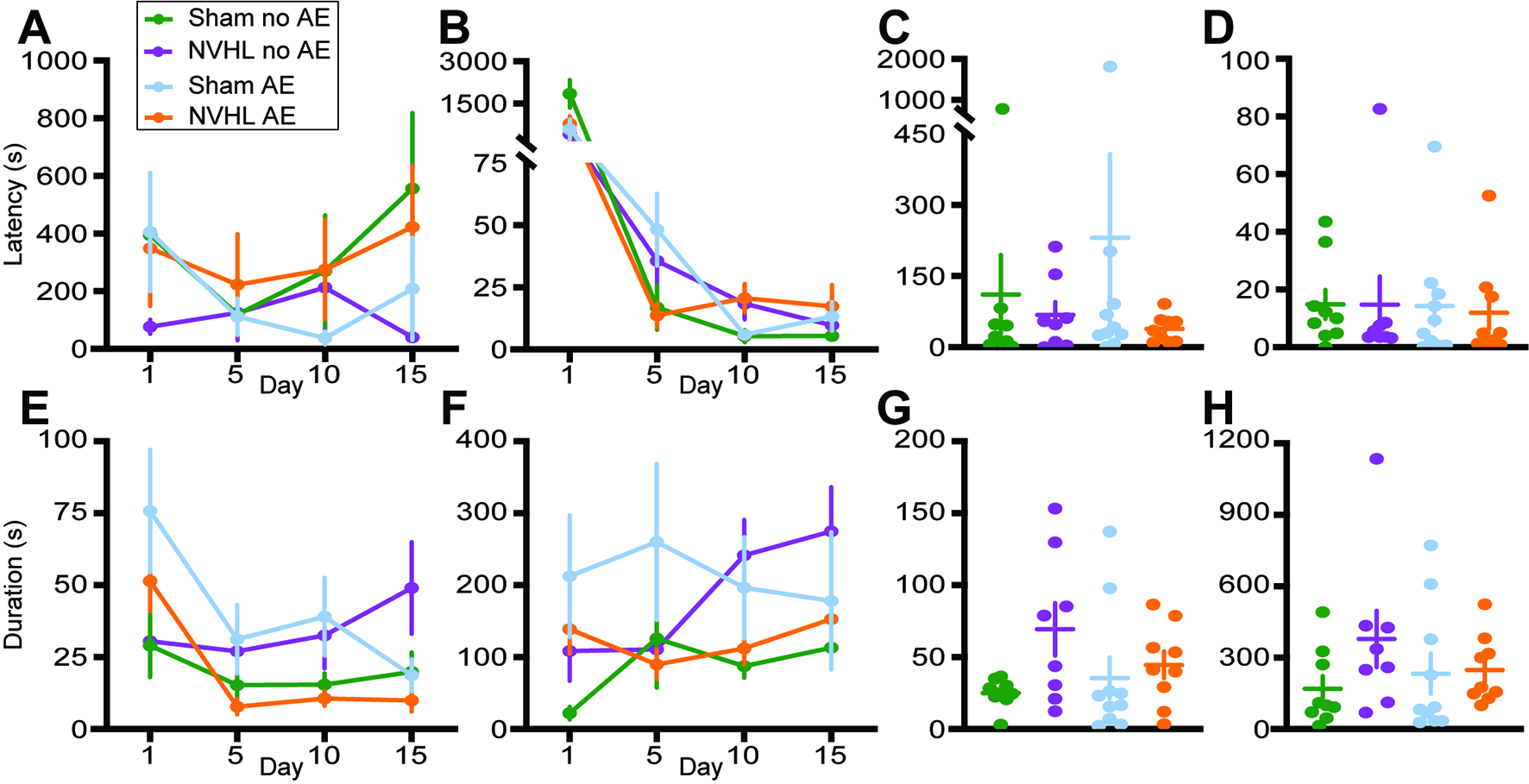

Supplement: Supplementary Figure 1 — Latency to and duration in center zone. (A) There were no significant group differences in latency to center zone during the preinjection phase on days 1, 5, 10, and 15. (B) There were no significant group differences in latency to center zone during the postinjection phase on days 1, 5, 10, and 15. (C) There were no significant group differences in latency to center zone during preinjection phase on the challenge day. (D) There were no significant group differences in latency to center zone during postinjection phase on the challenge day. (E) There were no significant group differences in the total duration of time spent in the center zone during the preinjection phase on days 1, 5, 10, and 15. (F) There were no significant group differences in the total duration of time spent in the center zone during the postinjection phase on days 1, 5, 10, and 15. (G) There were no significant group differences in the total duration of time spent in the center zone during the preinjection phase of the challenge day. (H) There were no significant group differences in the total duration of time spent in the center zone during the postinjection phase of the challenge day. Data is shown as group mean ± SEM. [file Image_1.TIF]
